# Respiratory microbiota diversity and composition in recurrent protracted bacterial bronchitis: a cross-sectional study

**DOI:** 10.3389/fcimb.2025.1524116

**Published:** 2025-07-28

**Authors:** Lidan Xu, Xiumei Ji, Mali Lin, Xipo Chen, Chan Su

**Affiliations:** Department of Pediatrics, Jinhua Maternal and Child Health Care Hospital, Jinhua, China

**Keywords:** lung microbiota, children, dysbiosis, microbial imbalance, recurrent protracted bacterial bronchitis

## Abstract

**Introduction:**

Recurrent protracted bacterial bronchitis (RPBB) is a significant risk factor for bronchiectasis in children, characterized by multiple episodes of protracted bacterial bronchitis (PBB) annually. With an increasing global incidence, a detailed understanding of RPBB’s pathophysiology is essential, particularly regarding the role of lung microbiota.

**Methods:**

This cross-sectional study recruited 39 children from Jinhua Maternal and Child Health Hospital between January 2021 and December 2022, including 18 with PBB, 11 with RPBB, and 10 as controls. Bronchoscopy with bronchoalveolar lavage (BAL) was performed to collect lung microbiota samples, which were analyzed using 16S rDNA sequencing. Microbial diversity and composition differences among groups were assessed using alpha and beta diversity metrics, PERMANOVA, and Linear Discriminant Analysis Effect Size (LEfSe), with statistical significance set at *P* < 0.05.

**Results:**

RPBB patients exhibited a distinct lung microbiota composition compared to controls, characterized by an increased abundance of pathogens such as *Acinetobacter* and *Mycoplasma*, alongside a reduction in beneficial genera like *Streptococcus* and *Granulicatella*. The RPBB group also demonstrated greater overall microbiota diversity, indicating dysbiosis that may contribute to disease severity and persistent respiratory symptoms.

**Conclusion:**

This study revealed significant alterations in the lung microbiota of children with RPBB, suggesting that microbial imbalance could play a crucial role in disease pathogenesis. These findings highlight the importance of targeted prevention and therapeutic strategies aimed at restoring microbiota balance to improve pediatric respiratory health.

## 1 Introduction

Recurrent protracted bacterial bronchitis (RPBB) is a recognized risk factor for bronchiectasis in children, characterized by protracted bacterial bronchitis (PBB) episodes occurring more than three times annually (Gallucci et al., 2020; Ruffles et al., 2021). The rising global incidence of RPBB highlights the urgent need for a deeper understanding of its pathophysiology to inform effective intervention strategies (Parisi et al., 2022). While studies have established that the lungs harbor a complex microbiome that plays a crucial role in respiratory health (Dickson and Huffnagle, 2015), the exact relationship between RPBB and lung microbiota remains poorly understood.

In healthy individuals, the lung microbiota contributes to airway homeostasis by reinforcing the epithelial barrier (Invernizzi et al., 2020), modulating the immune response (Ancona et al., 2023), and suppressing pathogens (Chen et al. (2020). Disruption of this microbial balance can lead to chronic inflammation, initiating PBB and, with persistence, RPBB (Marsh et al., 2019). Although PBB has been associated with structural changes in the lung microbiome (Atto et al., 2023; Everard et al., 2024), the microbial signatures of RPBB and their involvement in disease progression remain unclear.

Given that lung microbiota dysbiosis has been implicated in various chronic respiratory conditions, understanding its specific alterations in RPBB is crucial. However, the precise microbial changes associated with RPBB and their contribution to disease persistence remain undefined. To address this gap, this study aims to characterize and compare the lung microbiota in children with RPBB, PBB, and a control group (CG), providing insights into microbial imbalances that may drive RPBB pathogenesis. This study hypothesized that children with RPBB exhibited distinct lung microbiota alterations compared with those with PBB and healthy controls, characterized by increased microbial dysbiosis, including an overrepresentation of pathogenic taxa and a reduction in beneficial commensals. Furthermore, these microbial imbalances may contribute to RPBB pathogenesis and disease persistence.

## 2 Materials and methods

### 2.1 Study population

Children aged 6 months to 14 years were evaluated by experienced pediatric respiratory specialists through clinical interviews, physical examinations, and diagnostic tests—including chest imaging and echocardiography—to determine eligibility based on predefined inclusion and exclusion criteria. The inclusion criteria were as follows: For PBB, participants were required to meet all of the following: (1) Wet cough persisting for over 4 weeks; (2) Positive bacterial culture from sputum or bronchoalveolar lavage fluid (BALF) with a colony count ≥ 10^4^ CFU/mL; (3) Significant improvement following >2 weeks of antibiotic treatment; and (4) Exclusion of other causes of chronic cough. For RPBB, participants were required to meet both of the following: (1) Recurrent PBB episodes (>3 per year); and (2) Exclusion of other chronic cough causes. For CG (control group), participants had to meet both of the following: (1) Previously healthy; and (2) Diagnosed with an airway foreign body within 24 hours. Children with any of the following conditions associated with chronic cough, such as hemoptysis, growth retardation, congenital heart disease, neurological abnormalities, chronic rhinosinusitis, immunodeficiencies, tuberculosis, or chest wall deformities—were excluded from the study. This study was approved by the Jinhua Maternal and Child Health Hospital Ethics Committee [Approval No. 2021 (076)], and informed consent was obtained from all patients’ guardians.

A total of 39 children were enrolled between January 2021 and December 2022 at Jinhua Maternal and Child Health Hospital. The study cohort comprised 18 children with PBB (PBB group), 11 with RPBB (RPBB group), and a CG of 10 children with an acute tracheal foreign body (CG group). All participants were Han Chinese and resided in the same city. Peripheral blood and BALF samples were analyzed to assess immunoglobulin levels (IgA, IgG, and IgM), lymphocyte subsets (CD3^+^, CD4^+^, CD8^+^ T cells, and B cells), and BALF cellularity (total and differential cell counts). These parameters were measured to explore potential immunological differences among PBB, RPBB, and CG groups. Due to limited data on lung microbiome differences between RPBB patients and healthy subjects, an *a priori* sample size calculation was not feasible.

### 2.2 Bronchoscopy procedure

All participants underwent bronchoscopy, preceded by standard pre-procedure preparations, including routine blood tests, coagulation function assessments, pre-transfusion screening, and chest computed tomography (CT). Patients were required to fast for at least 4 hours before the procedure. Before the examination, midazolam (0.15 mg/kg, maximum dose 5 mg) was administered intravenously for sedation. The bronchoscope was inserted through the nasal or oral route up to the glottis. To provide local anesthesia, 1–2 mL of 1% lidocaine was sprayed onto the larynx and surrounding tissues through the biopsy channel. The bronchoscope was then advanced into the main trachea, where an additional 1–2 mL of 1% lidocaine was applied at the carina before accessing the left and right main bronchi.

In the PBB and RPBB groups, BALF was collected after assessing for airway abnormalities, while in the CG group, BALF was obtained following the removal of the foreign body. A 1 mL/kg aliquot of saline, pre-warmed to 37°C, was instilled into the bronchi of the right middle lobe and left lingula, and the lavage fluid was carefully aspirated. BALF samples were immediately sent for bacterial culture, cytological examination(quantifying total and differential cell counts including neutrophils, macrophages and lymphocytes), and inflammatory marker analysis, with additional aliquots stored at -80°C for future analyses. To minimize contamination, all bronchoscopic procedures were conducted using a disinfected bronchoscope, and sterile saline was used for bronchoalveolar lavage. BALF samples were collected using aseptic techniques and immediately processed to prevent external contamination.

### 2.3 Experimental methods and workflow

DNA was extracted from bronchoalveolar lavage fluid (BALF) samples using the QIAamp DNA Mini Kit (Qiagen, Germany) following the manufacturer’s protocol, with modifications to optimize bacterial DNA yield. Extracted DNA was quantified using a Qubit Fluorometer (Thermo Fisher Scientific, USA), and integrity was assessed by agarose gel electrophoresis. The V3-V4 hypervariable regions of the bacterial 16S rRNA gene were amplified using the universal primers 341F (5’-CCTACGGGNGGCWGCAG-3’) and 806R (5’-GGACTACHVGGGTATCTAAT-3’). Polymerase chain reaction (PCR) products were purified using AMPure XP magnetic beads (Beckman Coulter, USA) and pooled in equimolar concentrations for library construction. The 16S rDNA sequencing of all samples was performed by Hangzhou Jingyi Technology Co., Ltd., Hangzhou, China. The workflow included sample freezing, aliquoting, nucleic acid extraction, quality control, PCR amplification, purification of PCR products, library preparation, validation, and sequencing on the Illumina MiSeq platform. More specifically, sequencing was performed on the Illumina MiSeq platform using a 2 × 250 bp paired-end strategy. The sequencing depth was set to achieve an average of 50,000 reads per sample to ensure adequate microbial diversity characterization. Sequencing data were subjected to taxonomic composition analysis, diversity assessment, differential and correlation analysis, and statistical processing. For taxonomic classification, sequencing reads were processed using QIIME 2 (version 2022.2). The DADA2 plugin was used for quality filtering, denoising, chimera removal, and amplicon sequence variant (ASV) generation. Taxonomic assignment was performed using a Naïve Bayes classifier trained on the SILVA 138.1 SSURef NR99 database, which reflects the updated taxonomy based on the 2021 reclassification of bacterial and archaeal phyla (Oren and Garrity, 2021). A confidence threshold of 0.8 was applied. This version incorporates the latest taxonomic nomenclature updates in accordance with current microbial taxonomy standards, including the following reclassifications: Firmicutes (now Bacillota), Proteobacteria (now Pseudomonadota), Actinobacteria (now Actinomycetota), and Bacteroidetes (now Bacteroidota). All downstream taxonomic reporting, including genus- and phylum-level names in tables and figures, adheres to this updated classification system. Alpha and beta diversity metrics were computed within the QIIME 2 environment before further statistical analyses.

Serum immunoglobulin levels (IgA, IgG, and IgM) were measured using an immunoturbidimetric assay. Lymphocyte subsets were analyzed via flow cytometry to quantify CD3^+^, CD4^+^, CD8^+^ T cells, and B cells. BALF differential cell counts were performed using cytospin preparations and Wright-Giemsa staining.

Negative controls were included during DNA extraction and sequencing to identify potential contaminant sequences. Additionally, bioinformatics filtering was applied to remove known environmental and reagent-derived contaminants, ensuring that the microbiota profiles represented true biological variations.

To ensure consistency with the most recent microbial taxonomy, all taxonomic labels and classifications used throughout the study—particularly in **Figures 1**–**10** and **Table 1**—were aligned with the SILVA 138.1 database, incorporating the reclassifications recommended in the 2021 consensus report (Oren and Garrity, 2021).

**Figure 1:**
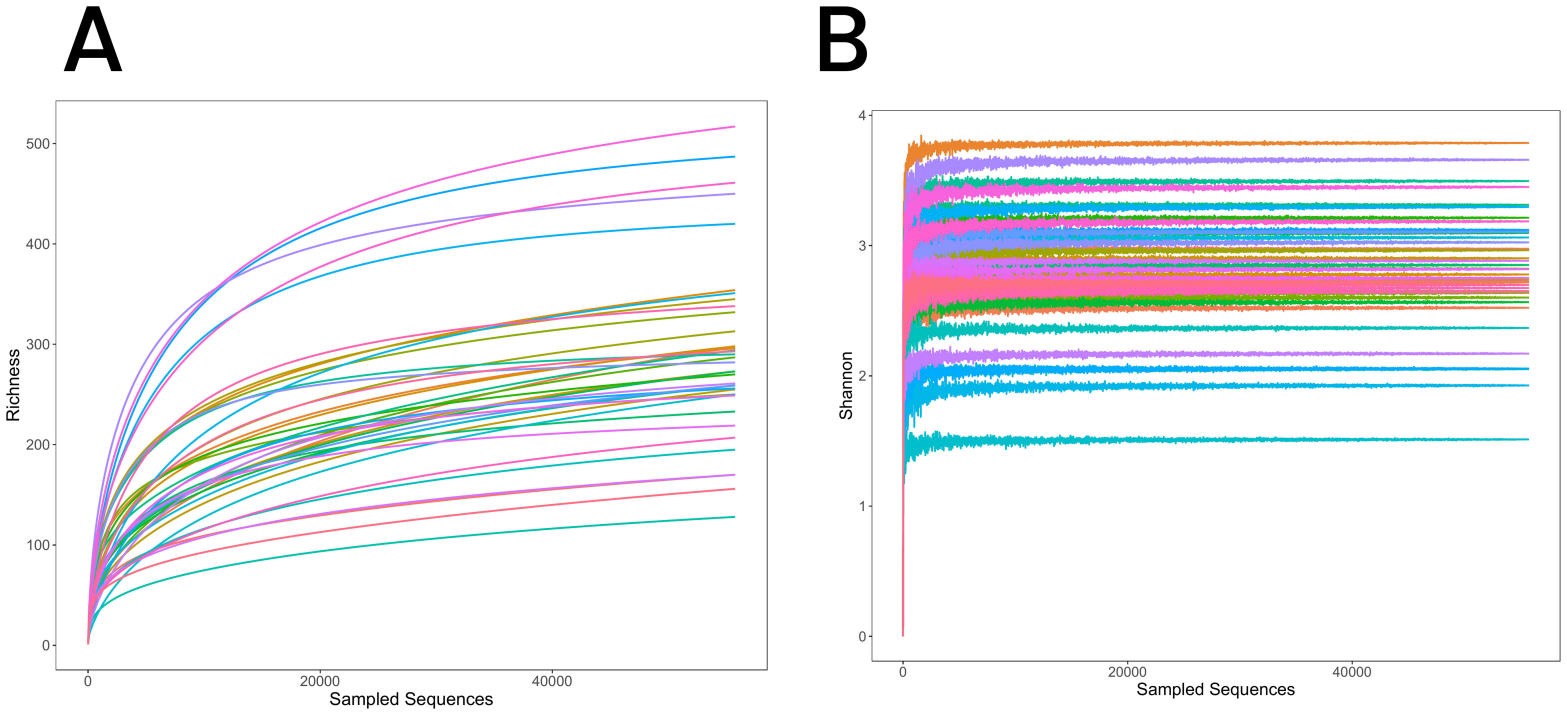
**(A)** Rarefaction curves for the three groups, illustrating species richness and sequencing depth. The curves plateau, indicating sufficient sequencing depth to capture the microbial diversity. **(B)** Shannon-Wiener diversity curves for the three groups, showing species diversity within each group. The curves level off with increasing sequencing depth, further confirming sequencing adequacy.

**Figure 2:**
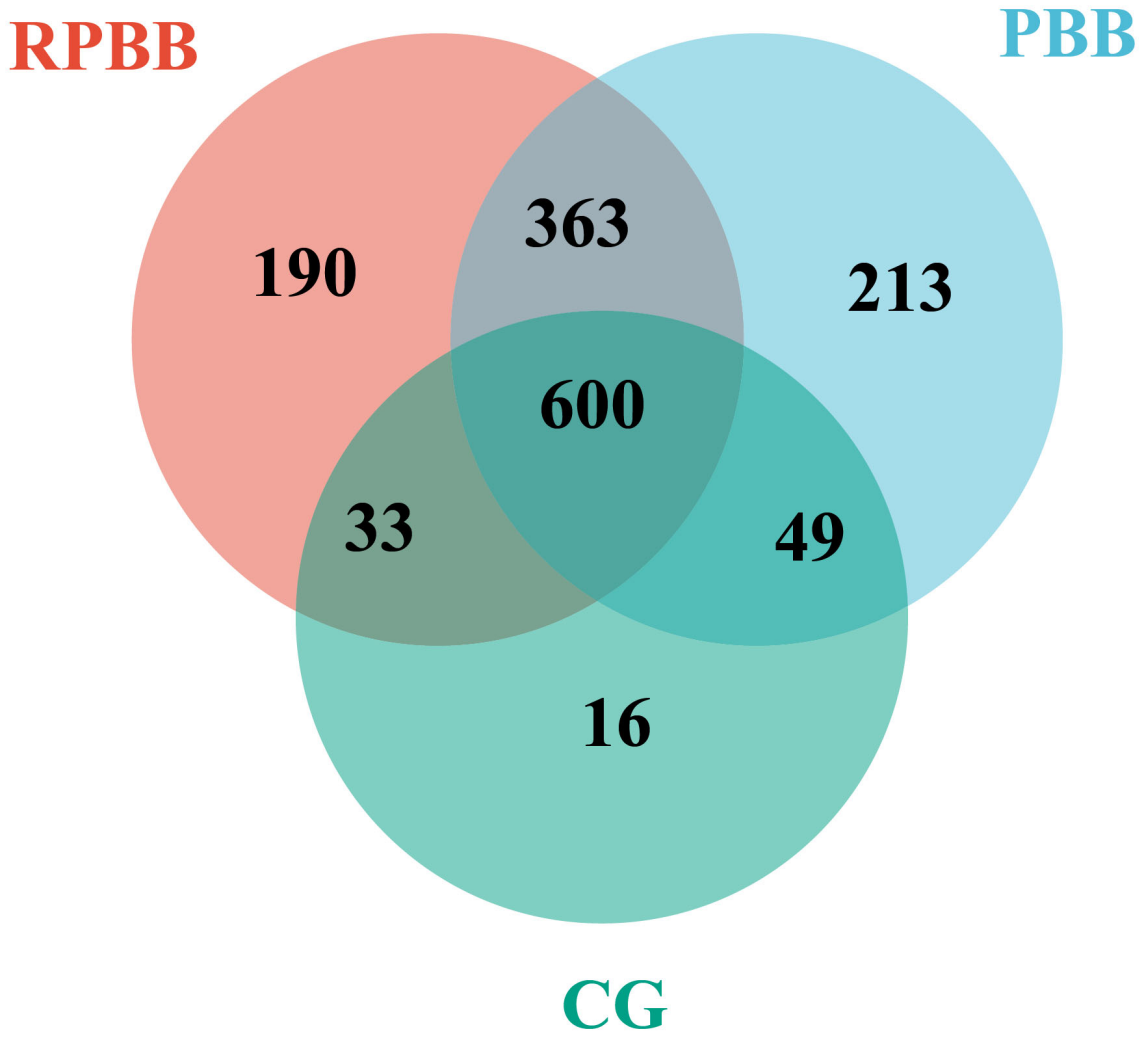
Venn diagram depicting the shared and unique bacterial species among the three groups. The RPBB and CG groups shared 633 species, with 553 unique to RPBB and 65 to CG. RPBB and PBB shared 963 species, while 223 were unique to RPBB and 262 to PBB. These findings suggest a substantial overlap in microbiota composition, with distinct species contributing to RPBB and PBB.

**Figure 3:**
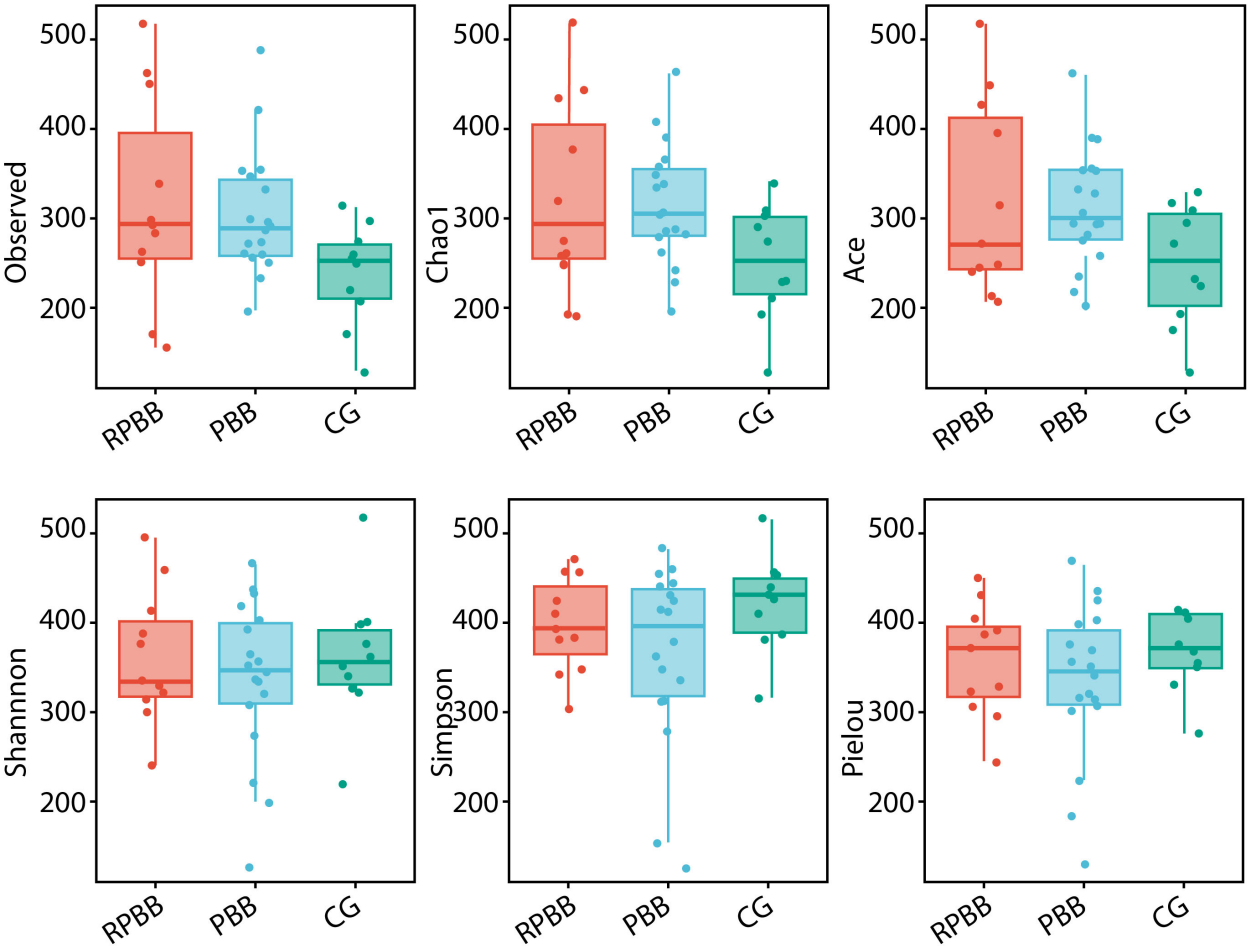
Alpha diversity indices (ACE, Chao1, Shannon, Observed species, Pielou, and Simpson) comparing species richness and evenness across the three groups. The RPBB group showed higher species richness values compared to the PBB and CG groups, while the CG group demonstrated greater species evenness than both RPBB and PBB groups. Each index is represented as a box plot to facilitate comparison of the observed trends.

**Figure 4:**
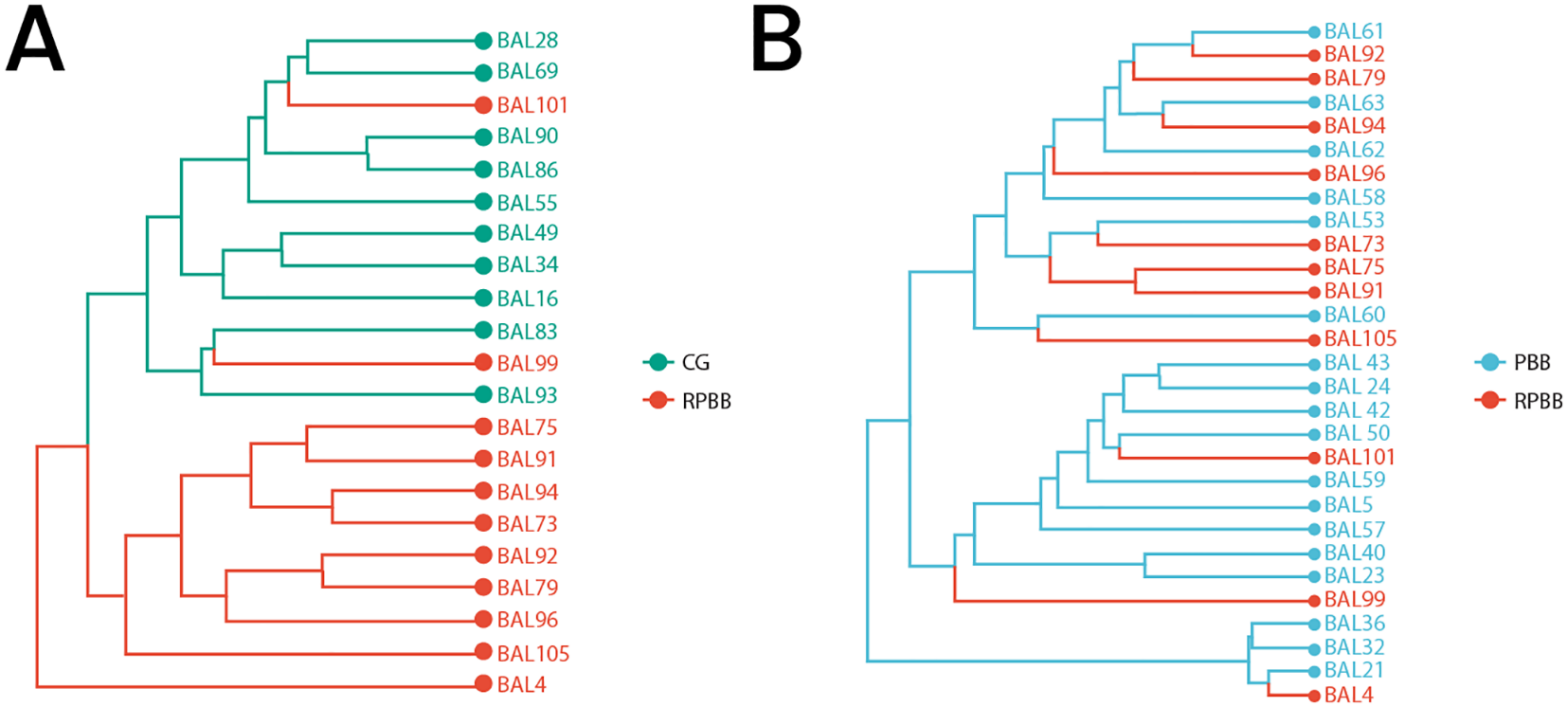
Beta diversity among the three groups as demonstrated through Hierarchical Clustering Analysis. **(A)** Cluster tree showing distinct separation between CG and RPBB, indicating significant differences in microbial composition. **(B)** Cluster tree illustrating the relationship between RPBB and PBB, where RPBB clusters within PBB, suggesting a microbiota shift potentially linked to disease progression.

**Figure 5:**
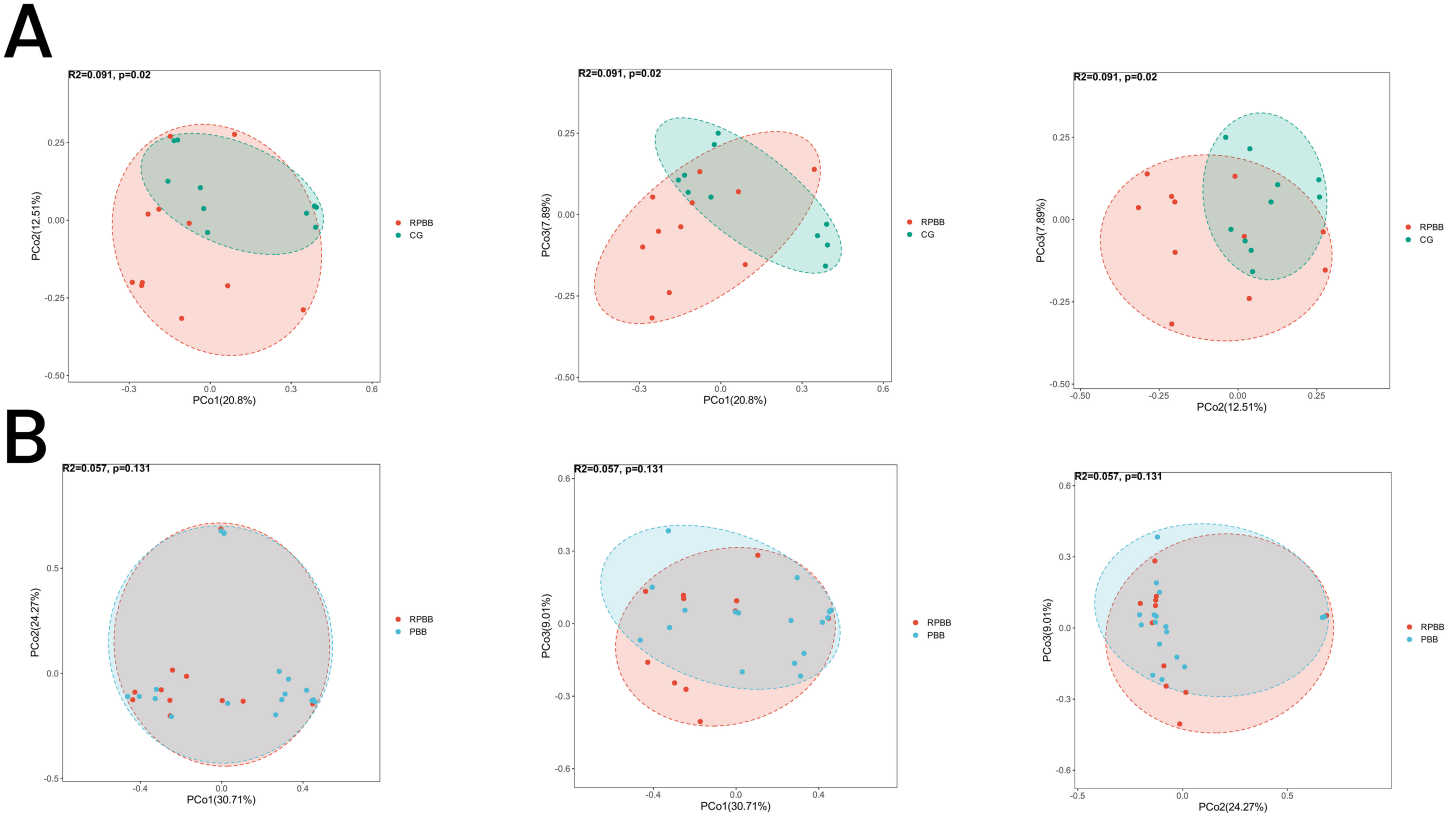
PCoA analysis illustrating beta diversity across the three groups. **(A)** The RPBB and CG groups formed distinct clusters, reflecting significant compositional differences in lung microbiota (*P* < 0.05). **(B)** The RPBB and PBB groups showed partial overlap, indicating some similarities, but overall compositional differences were not statistically significant (*P* > 0.05).

**Figure 6:**
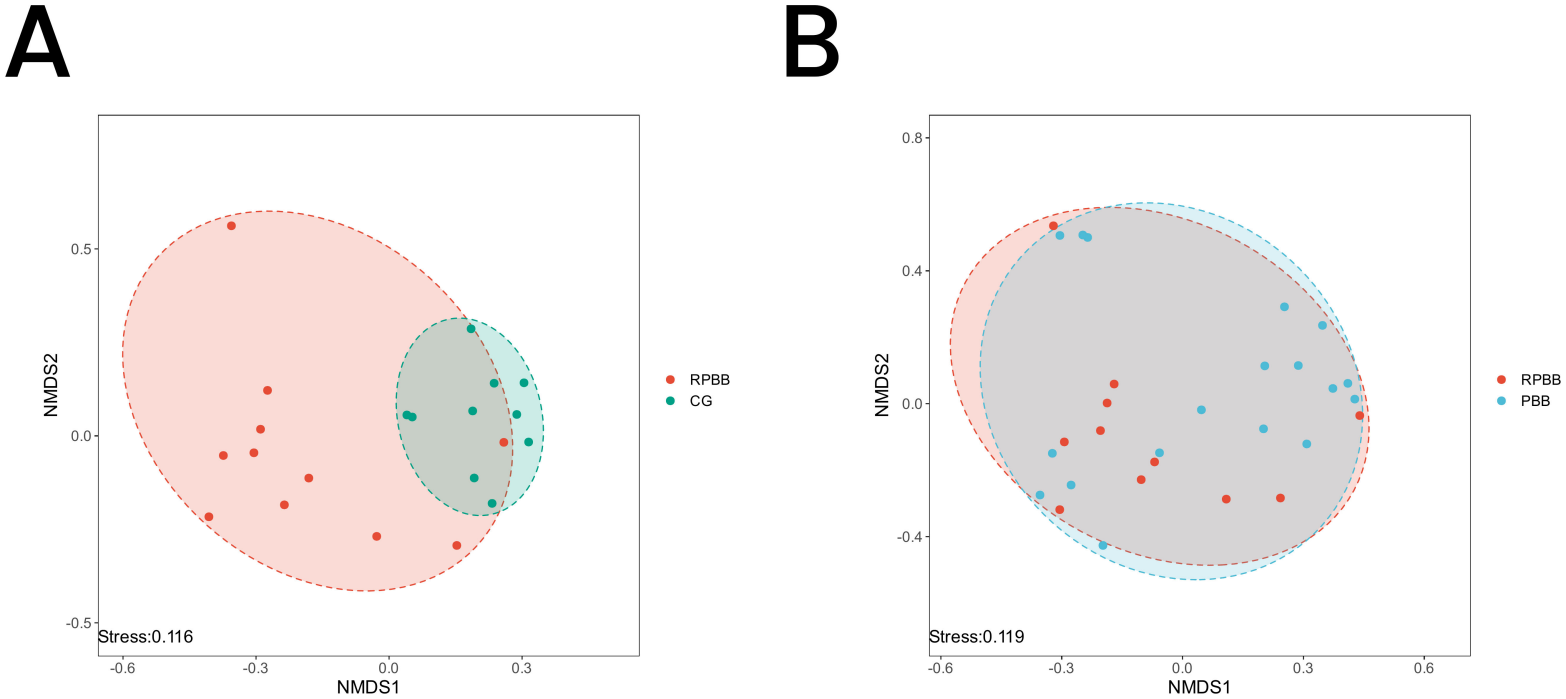
NMDS analysis depicting microbial community differences among the three groups. A stress value < 0.2 indicates a reliable representation of beta diversity. **(A)** RPBB and CG showed distinct clustering patterns, suggesting significant differences in microbiota composition. **(B)** RPBB and PBB exhibited partial overlap, reflecting both shared and unique microbiota characteristics.

**Figure 7:**
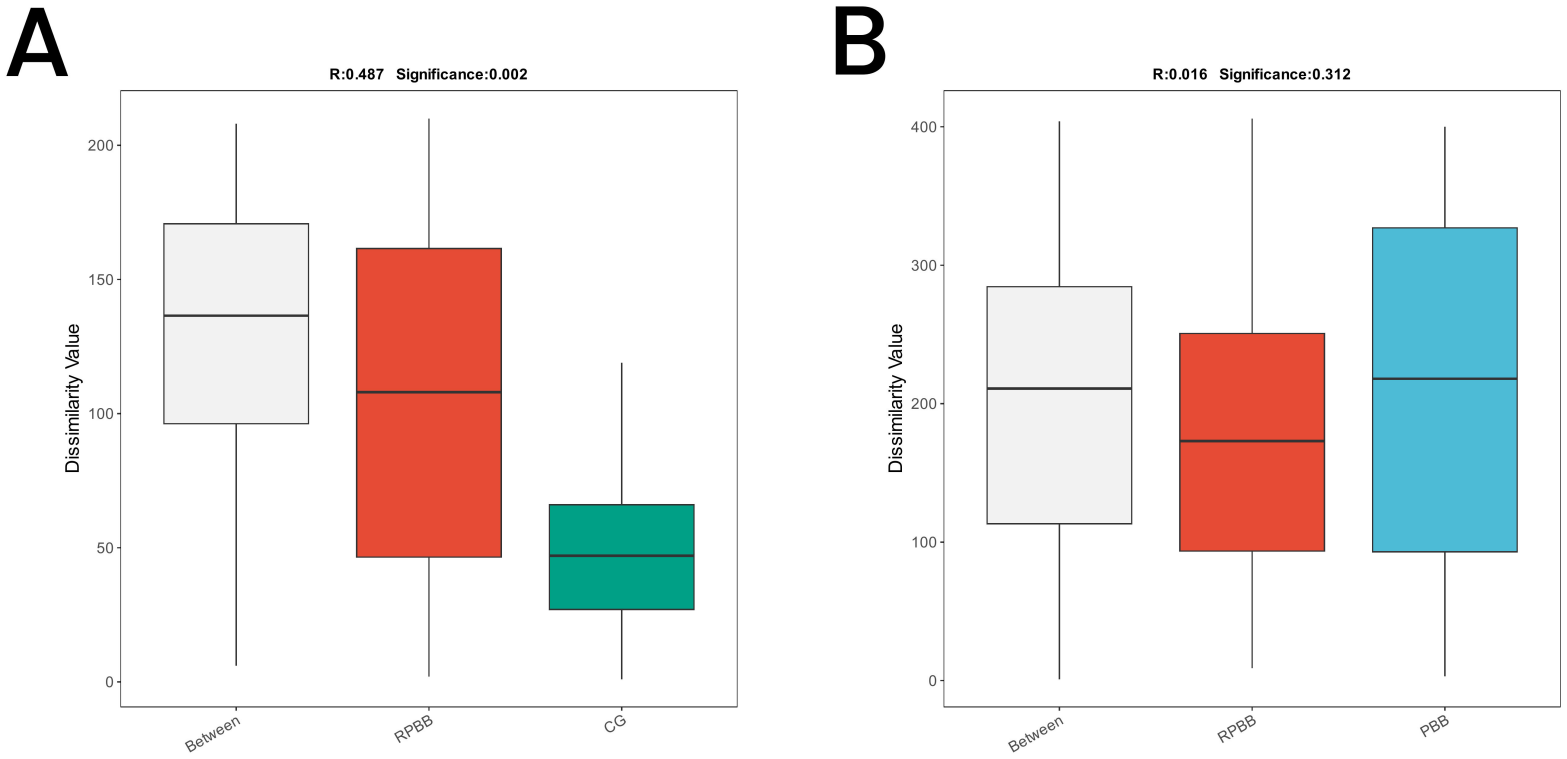
ANOSIM plots demonstrating beta diversity among the three groups. **(A)** ANOSIM analysis showed significant differences in microbial community structure between RPBB and CG (*R* = 0.487, *P* = 0.002), with greater variability in RPBB. **(B)** The differences between RPBB and PBB were not statistically significant (*P* > 0.05), suggesting similar microbial community structures.

**Figure 8:**
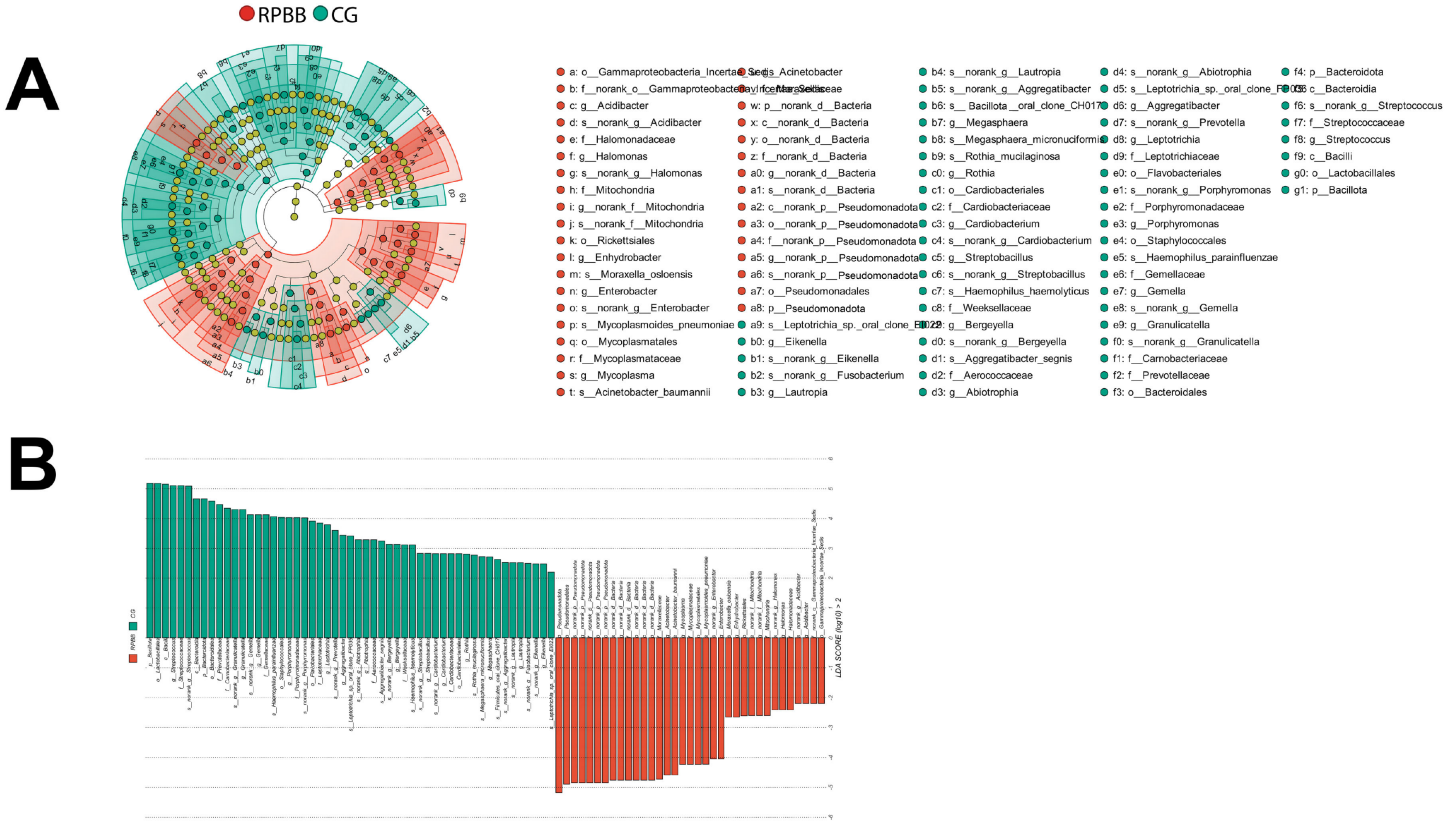
LEfSe analysis identifying differentially abundant taxa in the BALF samples. **(A)** Cladogram highlighting key bacterial taxa with significant differences between RPBB and CG groups. Red nodes represent taxa enriched in RPBB, while green nodes indicate those more abundant in CG. Yellow nodes represent taxa without significant differences. **(B)** Linear Discriminant Analysis (LDA) scores demonstrating statistically significant bacterial differences between RPBB and CG, with an LDA score threshold of 2.0 (*P* < 0.05). Taxonomic assignments follow the IJSEM 2021 taxonomy updates.

**Figure 9:**
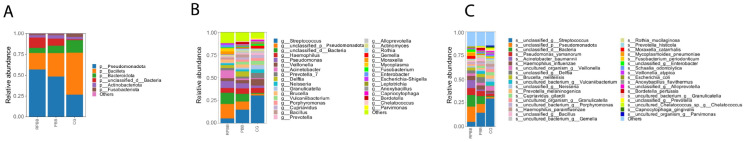
Bar diagram illustrating microbial community composition at the phylum, genus, and species levels. **(A)** At the phylum level, RPBB had a significantly lower abundance of Bacillota compared to CG (*P* < 0.05), while Pseudomonadota were significantly enriched (*P* < 0.05), indicating a shift in microbial balance. **(B, C)** At the genus and species levels, RPBB showed significantly increased levels of gram-negative bacteria, including *Acinetobacter*, *Pseudomonas*, and *Hemophilus*, while beneficial genera such as *Streptococcus* were reduced (*P* < 0.05). Taxonomic assignments reflect the reclassification by Oren and Garrity (2021).

**Figure 10:**
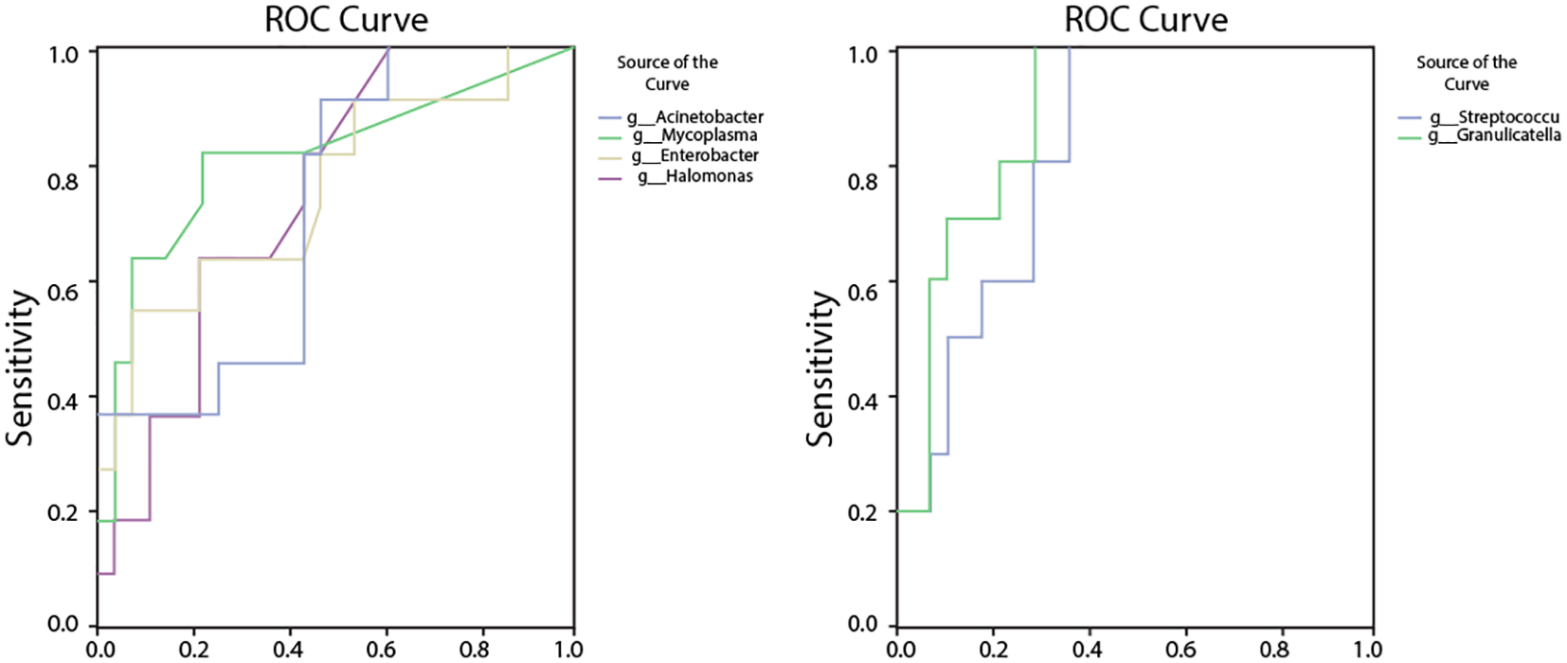
ROC analysis assessing the diagnostic potential of bacterial genera with significantly altered abundances in RPBB. **(A)** ROC curves for bacteria enriched in RPBB, including *Acinetobacter* (AUC = 0.79, *P* = 0.024), *Mycoplasma* (AUC = 0.86, *P* = 0.006), and *Enterobacter* (AUC = 0.77, *P* = 0.038). **(B)** ROC curves for bacterial genera with significantly lower abundances in RPBB, such as *Streptococcus* (AUC = 0.96, *P* < 0.001) and *Granulicatella* (AUC = 0.964, *P* < 0.001), which exhibited high sensitivity and specificity as potential diagnostic markers.

**Table 1:** Microbiota composition at the phylum level in three groups.

Feature	RPBB	CG	Z-value	*P*-value
p_Pseudomonadota	0.569	0.268	4.31	<0.001
p_Bacillota	0.198	0.503	-4.61	<0.001
p_Bacteroidota	0.059	0.151	-2.36	0.018
p_unclassified_d:Bacteria	0.124	0.009	3.57	<0.001
p_Actinobacteriota	0.030	0.030	0.45	0.651
p_Fusobacteriota	0.0137	0.034	-1.01	0.312
Others	0.006	0.005		

### 2.4 Statistical analysis

Data analysis was performed using SPSS (version 22.0), QIIME 2 (version 2022.2), and R (version 3.6.1) software. Quality control and normalization were performed before statistical analyses. Alpha diversity was assessed using Shannon and Chao1 indices, and differences in alpha diversity among groups were evaluated using the Kruskal-Wallis test, followed by *post hoc* Dunn’s test for pairwise comparisons. Beta diversity was calculated via the Bray-Curtis distance matrix and visualized with Principal Coordinates Analysis (PCoA). Group differences were statistically assessed using Permutational Multivariate Analysis of Variance (PERMANOVA) to determine significant differences between microbiota compositions. Quantitative data are presented as medians with interquartile ranges (IQR) and categorical data as frequencies and percentages. Relative genus abundances across groups were analyzed using Linear Discriminant Analysis Effect Size (LEfSe). Intergroup differences in bacterial relative abundances were analyzed using the Kruskal-Wallis test for multiple-group comparisons, followed by the Wilcoxon rank-sum test for pairwise comparisons. Multiple hypothesis testing was corrected using the Benjamini-Hochberg false discovery rate (FDR) method. Diagnostic potential for specific bacterial species was evaluated using receiver operating characteristic (ROC) curves generated with the ‘pROC’ and ‘ggplot2’ R packages. Taxa included in the ROC curve analysis were selected based on their differential abundance across groups and potential relevance to RPBB pathogenesis, rather than solely on the highest effect size in the LDA analysis. The selection process considered both statistical significance and clinical relevance, ensuring that the analyzed taxa had potential diagnostic value. Statistical significance was set at *P* < 0.05.

## 3 Results

### 3.1 Clinical features and BALF pathogen profiles in RPBB and PBB patients

In the RPBB group (*n* = 11; 4 male, 7 female; median age: 7 years; range: 2–11 years), the disease duration ranged from 1 to 3 years (**Table 2**). Three patients had wheezing, three had eczema, and all had received ineffective inhaled corticosteroids along with prior antibiotic treatments, primarily oral cephalosporins, macrolides, and amoxicillin, often administered for over 10 days in multiple courses. BALF cultures were positive in five cases, identifying *Moraxella catarrhalis* (1), *Streptococcus pneumoniae* (3), and *Staphylococcus aureus* (1). In the PBB group (*n* = 18; 9 male, 9 female; median age: 4 years 4 months; range: 1 year 4 months–7 years), disease duration ranged from 1 to 6 months. Wheezing was reported in eight patients, while seven had eczema. Eighteen patients had received prior antibiotic treatments, primarily cephalosporins, macrolides, and amoxicillin, for over 10 days. BALF cultures were positive in four cases, detecting adenovirus (1), *Streptococcus pneumoniae* (1), *Streptococcus mitis* (1), and *Bordetella pertussis* (1). In the CG group(n = 10; 7 male, 3 female; median age: 1 years 2 months; range: 8months-2 years 4 months), BALF cultures were negative in all cases, and no abnormalities in humoral or cellular immunity were found across groups.

**Table 2:** Summarizing the characteristics of participants included in the study.

Characteristic	RPBB Group (n=11)	PBB Group (n=18)	CG Group (n=10)
Sex (Male/Female)	4/7	9/9	7/3
Median Age (Years, Range)	7 (2–11)	4.3 (1.3–7)	1.2(0.7-2.3)
Disease Duration	1–3 years	1–6 months	Acute (foreign body)
Wheezing (n, %)	3 (27%)	8 (44%)	0 (0%)
Eczema (n, %)	3 (27%)	7 (39%)	0 (0%)
Prior Antibiotic Use (n, %)	11 (100%)	18 (100%)	0 (0%)
Common Antibiotics Used	Cephalosporins, Macrolides, Amoxicillin	Cephalosporins, Macrolides, Amoxicillin	Not applicable
BALF Culture Positive (n, %)	5 (45%)	4 (22%)	0 (0%)
Identified Pathogens	M. catarrhalis (1), S. pneumoniae (3), S. aureus (1)	Adenovirus (1), S. pneumoniae (1), S. mitis (1), B. pertussis (1)	No pathogens detected

### 3.2 Rarefaction curve analysis

Rarefaction curves were used to evaluate sequencing depth by plotting the number of species detected relative to sample size. The curves plateaued as sample size increased, indicating that sequencing depth was sufficient, with minimal new OTUs identified beyond this point (**Figure 1A**). Similarly, the Shannon diversity index stabilized with increasing sequencing depth, further confirming the adequacy of the dataset in capturing microbial diversity across samples (**Figure 1B**). These findings support the reliability of sequencing depth for characterizing lung microbiota in the RPBB, PBB, and CG groups.

### 3.3 Similarity analysis of lung microbiota in RPBB, PBB, and CG groups

Venn diagram analysis demonstrated both shared and unique bacterial species among the groups. The RPBB and CG groups shared 633 species, while 553 species were unique to RPBB and 65 were unique to CG. Between RPBB and PBB, 963 species were shared, whereas 223 were unique to RPBB and 262 to PBB. These results suggest that while RPBB shares a substantial portion of its microbiota with CG and PBB, certain species are specific to RPBB. These unique species may play a role in the onset and progression of RPBB (**Figure 2**).

### 3.4 Alpha diversity analysis of lung microbiota in RPBB group

Alpha diversity analysis assessed species richness and evenness within each group. The analysis revealed that while the RPBB group showed numerically higher values for the “Observed,” “Chao1,” and “ACE” indices compared to both the PBB and control groups (CG), these differences did not reach statistical significance (P > 0.05). Similarly, comparisons of the Simpson index, Shannon index, and Pielou's evenness index among the three groups demonstrated no statistically significant variations (P > 0.05) (**Figure 3**). **Figure 3** presents box plots comparing alpha diversity indices across groups. Consistent trends were observed: the RPBB group showed elevated values in richness indices (Observed species, Chao1, ACE), while the CG group exhibited relatively higher values in diversity/evenness indices (Shannon, Simpson, Pielou). These patterns suggest potential ecological distinctions that warrant further exploration.

### 3.5 Beta diversity analysis of lung microbiota in RPBB group

#### 3.5.1 Hierarchical clustering analysis

Dendrogram analysis revealed distinct clustering patterns, with CG samples grouping at the top and RPBB samples clustering at the bottom, indicating distinct microbiota compositions in each group (**Figure 4A**). In another dendrogram, RPBB branched from within the PBB cluster, suggesting that RPBB may represent a variant or progression of PBB (**Figure 4B**).

#### 3.5.2 PCoA

PCoA was used to assess the diversity and similarity of lung microbiota among the RPBB, CG, and PBB groups. PCoA indicated a clear separation between the lung microbiota of RPBB and CG, forming distinct clusters that reflect significant compositional differences (**Figure 5A**). The CG group exhibited lower variability, whereas the RPBB group displayed greater diversity. While RPBB and PBB exhibited some compositional overlap, they still maintained distinct differences, consistent with phylogenetic tree analysis (**Figure 5B**).

#### 3.5.3 Non-metric multidimensional scaling analysis

The NMDS plot visualized similarities and differences in microbial community composition among the groups. The RPBB group exhibited a more dispersed clustering pattern compared to CG, suggesting greater diversity within RPBB samples. Despite some overlap, the distinct spatial distribution suggests unique microbiota characteristics in each group (**Figure 6A**). The RPBB and PBB groups showed partial overlap, reflecting both shared and distinct microbiota features (**Figure 6B**).

#### 3.5.4 Analysis of similarities

ANOSIM was conducted to determine whether differences between groups exceeded within-group variation, providing insight into community structure. The analysis confirmed significantly greater differences in microbial community composition between RPBB and CG (R = 0.487, *P* = 0.002), as shown in the box plots, with RPBB exhibiting higher internal variability. However, differences between RPBB and PBB were not statistically significant (**Figures 7A, B**).

### 3.6 LEfSe analysis

LEfSe was used to identify key differential taxa between groups, with the Linear Discriminant Analysis (LDA) score threshold set at 2.0. At the phylum level, the RPBB group exhibited a significant increase in Pseudomonadota and a marked decrease in Bacillota and Bacteroidota compared to the CG group. At the genus level, RPBB showed significantly higher abundances of *Acinetobacter, Mycoplasma, Enterobacter, Halomonas*, and *Acidibacter*, while beneficial genera such as *Streptococcus, Granulicatella, Gemella, Porphyromonas, Leptotrichia, Aggregatibacter*, and *Abiotrophia* were significantly lower than in the CG group (**Figures 8A, B**).

### 3.7 Major microbiota composition

To characterize the lung microbiota in RPBB patients, species composition analysis was conducted at the phylum, genus, and species levels. **Table 1** presents microbiota composition at the phylum level in three groups.

At the phylum level, Bacillota, Pseudomonadota, Bacteroidota, Actinobacteriota, and Fusobacteriota dominated the microbiota across all groups, although their relative abundance rates differed (**Table 1**). In CG, these phyla accounted for 98.56% of the microbiota, leaving only 1.44% as non-core bacteria. In contrast, RPBB exhibited a lower proportion of core phyla (86.97%) and a higher presence of non-core bacteria (13.03%), suggesting increased microbial diversity. Bacillota were significantly less abundant in RPBB (19.76%) than in CG (50.25%) (*P* < 0.05), whereas Pseudomonadota were markedly increased (56.9% in RPBB vs. 26.8% in CG). This shift suggests a microbial imbalance, with an enrichment of gram-negative bacteria in RPBB.

At the genus level, RPBB and PBB exhibited similar dominant genera compared to CG. Beneficial genera such as *Streptococcus*, *Veillonella*, *Prevotella*, and *Bifidobacterium* were more abundant in CG but showed reductions in RPBB. Conversely, RPBB displayed higher levels of gram-negative genera, including *Acinetobacter*, *Pseudomonas*, and *Hemophilus* (**Figure 9B**).

At the species level, RPBB patients exhibited significantly increased abundance rates of *Acinetobacter baumannii*, *Hemophilus influenzae*, *Pseudomonas aeruginosa*, and *Escherichia coli*, which are known respiratory pathogens (**Figure 9C**). These taxa, although not necessarily having the highest LDA effect sizes in the LEfSe analysis, were selected based on their established clinical relevance and significant differences between groups.

### 3.8 ROC curve analysis of key bacterial genera

The relative abundance rates of *Acinetobacter*, *Mycoplasma*, *Enterobacter*, *Halomonas*, *Acidibacter*, *Streptococcus*, and *Granulicatella* were found to be associated with RPBB. Receiver operating characteristic (ROC) analysis was performed to evaluate their diagnostic potential at the genus level. *Acinetobacter* had an AUC of 0.79 (95% CI: 0.59–0.99, *P* = 0.024), with a cut-off threshold of 0.332, yielding a sensitivity of 0.91 and a specificity of 0.70. *Mycoplasma* had an AUC of 0.86 (95% CI: 0.68–1.00, *P* = 0.006), with a cut-off threshold of 0.0072, yielding a sensitivity of 0.82 and a specificity of 0.60. *Enterobacter* had an AUC of 0.77 (95% CI: 0.56–0.97, *P* = 0.038), with a cut-off threshold of 0.0762, yielding a sensitivity of 0.82 and a specificity of 0.80. *Halomonas* had an AUC of 0.82 (95% CI: 0.62–1.00, *P* = 0.014), with a cut-off threshold of 0.0045, yielding a sensitivity of 0.82 and a specificity of 0.80. *Acidibacter* had an AUC of 0.76 (95% CI: 0.56–0.92, *P* = 0.041), with a cut-off threshold of 0.0081, yielding a sensitivity of 0.64 and a specificity of 0.80. *Streptococcus* had a high AUC of 0.96 (95% CI: 0.86–1.00, *P* < 0.001), with a cut-off threshold of 8.1842, yielding a sensitivity of 1.00 and a specificity of 0.91. *Granulicatella* had an AUC of 0.96 (95% CI: 0.89–1.00, *P* < 0.001), with a cut-off threshold of 0.0045, yielding a sensitivity of 1.00 and a specificity of 0.91 (**Figure 10**).

### 3.9 BALF cellularity and differential cell counts

Total BALF cell counts were significantly higher in the RPBB group compared to the PBB and CG groups (median [IQR]: 8.2 [6.9–9.5] × 10^6^ cells/mL vs. 5.6 [4.2–6.8] × 10^6^ cells/mL vs. 2.9 [2.1–3.5] × 10^6^ cells/mL, *P* < 0.001). Differential cell counts revealed a predominance of neutrophils in RPBB (median: 72.5% [65.3–78.6]), whereas macrophages were the predominant cell type in CG (median: 80.2% [75.8–85.1]). The PBB group exhibited an intermediate cellular profile, with both neutrophils (45.7% [40.2–50.9]) and macrophages (50.1% [46.5–55.3]) present at comparable proportions.

### 3.10 Serum immunoglobulin levels

Serum IgA, IgG, and IgM levels did not differ significantly between groups (*P* > 0.05), suggesting no major systemic humoral immune dysfunction in RPBB or PBB. However, RPBB patients exhibited a trend toward lower IgG levels compared to CG (RPBB: 6.8 [5.9–7.5] g/L vs. CG: 8.2 [7.5–9.1] g/L, *P* = 0.07), which may indicate a compromised adaptive immune response.

### 3.11 Lymphocyte subset analysis

Flow cytometric analysis demonstrated a significantly lower CD4^+^/CD8^+^ ratio in the RPBB group compared to the CG group (median [IQR]: 1.15 [0.98–1.31] vs. 1.54 [1.42–1.69], *P* = 0.003), indicating a potential immune imbalance in RPBB. The proportion of CD3^+^ T cells was significantly lower in RPBB (55.8% [50.6–61.2]) than in CG (66.1% [62.5–70.4], *P* = 0.02). Similarly, CD4^+^ T cell frequencies were lower in RPBB (29.4% [27.1–32.8]) than in CG (38.5% [35.2–41.7], *P* = 0.01), while CD8^+^ T cells were relatively increased (25.7% [22.9–28.1] in RPBB vs. 23.1% [20.4–25.3] in CG, *P* = 0.04). B-cell frequencies did not differ significantly between groups (*P* > 0.05).

## 4 Discussion

The lungs, rather than being sterile, harbor a dynamic microbial community that plays essential roles in strengthening the airway epithelial barrier, regulating immune responses, and mitigating inflammation (Konovalovas et al., 2019). Beneficial bacteria typically suppress opportunistic pathogens, keeping them at low levels (Marathe et al., 2022). However, inflammation increases airway permeability, mucus accumulation, and the secretion of inflammatory factors, creating favorable conditions for pathogen proliferation and microbial dysbiosis (Budden et al., 2019). This study found that lung microbiota in the CG group was stable and diverse, whereas RPBB patients exhibited significant alterations, including increased bacterial abundance (**Figure 3**). Dendrogram and PCoA analyses revealed substantial differences in microbial community structure between the RPBB and CG groups (**Figures 4A**, **5A**). In contrast, dendrogram and NMDS plots showed that RPBB and PBB groups exhibited partial overlap, though with distinguishable microbial patterns (**Figures 4B**, **6B**). These findings are supported by the Venn diagram showing shared and unique bacterial species among the groups (**Figure 2**). The unique microbiota composition in RPBB patients and the elevated presence of non-endogenous bacteria likely indicate rapid proliferation following disease onset, disrupting pulmonary microecological balance. Further investigation into the dominant bacterial communities in RPBB patients is necessary, as they may play a key role in disease progression.

Alpha diversity indices showed trends of reduced microbial evenness and diversity in RPBB and PBB patients compared to CG ,though these differences did not reach statistical significance (**Figure 3**). Beta diversity analyses, including hierarchical clustering, PCoA, and NMDS, nevertheless confirmed distinct microbial compositions in RPBB versus CG, with the RPBB microbiota showing more dispersion and heterogeneity (**Figures 4**-**6**). ANOSIM results quantitatively confirmed these differences, with a statistically significant separation between RPBB and CG (**Figure 7A**), though no significant separation between RPBB and PBB (**Figure 7B**). These data indicate that while RPBB and PBB share features, RPBB may represent a progression with increased microbial disruption. LEfSe analysis identified differential taxa between groups. RPBB was characterized by an increased abundance of gram-negative genera, such as *Acinetobacter, Mycoplasma, Enterobacter, Halomonas*, and *Acidibacter*, and a significant depletion of beneficial genera, involving *Streptococcus* and *Granulicatella* (**Figure 8**). ROC curve analysis further demonstrated that several of these genera, particularly *Streptococcus* and *Granulicatella*, had strong discriminatory power in identifying RPBB patients (**Figure 10**).

The composition of the lower respiratory tract microbiota in children with RPBB differs significantly from that of healthy controls. Microbiota composition analysis at different taxonomic levels revealed that CG subjects had higher levels of Bacillota and beneficial genera, while RPBB samples were dominated by Pseudomonadota and respiratory pathogens, such as *Acinetobacter baumannii* and *Pseudomonas aeruginosa* (**Figure 9**). These compositional shifts suggest a transition from a balanced to a pathogen-dominated microbiota in RPBB, potentially contributing to recurrent infection and impaired resolution. This study identified notable differences in bacterial diversity, abundance, and composition between RPBB patients and healthy children, revealing unique microbial communities potentially associated with RPBB onset and progression. In healthy children, the predominant bacterial phyla in the lungs were Bacillota (50.25%) and Bacteroidota (15.12%). However, in RPBB patients, the proportion of Bacillota decreased to 19.76%, and Bacteroidota to 5.93% (**Table 1**). These phyla include symbiotic bacteria linked to immune regulation and anti-inflammatory responses, contributing to respiratory health (Ibironke et al., 2020; Paudel et al., 2020). Their reduction may lead to unchecked inflammation, potentially driving RPBB pathogenesis. In contrast, Pseudomonadota abundance in RPBB patients increased significantly from 26.8% to 56.9%, alongside an increase in other microbial taxa, collectively accounting for 13.03% (**Table 1**). This suggests a more complex respiratory microbiota enriched with Gram-negative pathogens in RPBB patients. Notably, microbial profiles in RPBB patients also differed from those observed in other respiratory diseases with similar symptoms. For instance, asthma patients typically exhibit a higher abundance of Actinobacteria and lower levels of Pseudomonadota (van Beveren et al., 2023; Valverde-Molina and García-Marcos, 2023). These microbial differences may provide valuable insights for the differential diagnosis of RPBB.

*Streptococcus* and *Granulicatella* are key components of the normal respiratory microbiota, playing a vital role in maintaining respiratory microbial homeostasis (Fabbrizzi et al., 2019). These genera limit pathogen colonization through competitive exclusion and the production of antimicrobial peptides while modulating the host immune response to enhance local immune defenses (Haldar et al., 2020; Narendrakumar and Ray, 2022). A reduction in these beneficial microbes disrupts microbial balance, lowering resistance to pathogens. Such dysbiosis may facilitate pathogen colonization and proliferation, potentially contributing to the onset of RPBB (Scialò et al., 2023). Previous studies have linked the decline in normal microbiota with increased susceptibility to respiratory infections, emphasizing their protective role in respiratory health (Zhou et al., 2024). Conversely, the increased abundance of Gram-negative pathogens such as *Acinetobacter*, *Mycoplasma*, *Enterobacter*, *Halomonas*, and *Acidibacter* in the lung microbiota of RPBB patients suggests their potential pathogenic roles in disease progression. These bacteria exhibit strong pro-inflammatory properties, which may exacerbate lung inflammation through airway microenvironment alterations, thereby worsening respiratory symptoms and contributing to RPBB development (Natalini et al., 2023). For instance, *Acinetobacter* is a well-known opportunistic pathogen with heightened pathogenicity in immunocompromised patients (Wang et al., 2023). The proliferation of *Mycoplasma* and *Enterobacter* may further aggravate RPBB symptoms by promoting inflammatory responses (Wang et al., 2021; Georgakopoulou et al., 2024). Additionally, *Halomonas*, typically found in marine environments, has been identified as an emerging opportunistic pathogen in immunocompromised individuals, warranting further investigation into its role in RPBB (Lu et al., 2022). Collectively, the integration of sequencing-based microbial profiling with immune and cellular analyses provides a comprehensive view of RPBB pathophysiology. The figure-supported findings underscore distinct microbiota alterations in RPBB, distinguishing it from PBB and CG, and point toward potential diagnostic biomarkers and therapeutic targets.

Furthermore, ROC curve analysis indicated that the relative abundance of these bacterial genera could serve as early diagnostic biomarkers for RPBB. This finding offers new possibilities for early and accurate RPBB diagnosis and lays the groundwork for developing personalized therapeutic strategies. Targeted interventions focusing on specific microbial genera may present a novel approach to RPBB treatment. Early identification of high-risk individuals could enable timely interventions, potentially mitigating disease severity and complications.

The frequent use of antibiotics before hospitalization in PBB and RPBB patients could influence the lung microbiota composition observed in this study. Numerous patients had undergone multiple courses of macrolides, cephalosporins, or amoxicillin, which could selectively reduce certain bacterial populations while promoting the persistence of antibiotic-resistant or opportunistic pathogens. Previous studies have suggested that repeated antibiotic exposure could lead to microbiome dysbiosis, characterized by decreased microbial diversity and alterations in community structure. This pre-hospital treatment variability could partly explain the observed differences in lung microbiota between RPBB, PBB, and CG groups, as well as the incomplete therapeutic response in some RPBB patients. Future studies should consider systematically recording outpatient antibiotic regimens and treatment durations to better assess their impact on bacterial imbalance and disease progression.

This study has several limitations. Its cross-sectional design limits the ability to establish causal relationships between microbiota composition and disease recurrence. Longitudinal studies are needed to track microbiota changes over time and clarify their role in disease progression or resolution. Additionally, the sampling method may not fully capture the dynamic variability of the lung microbiota, as samples were collected at a single time point. Environmental factors, such as recent antibiotic use or exposure to pollutants, may have influenced microbiota composition but were not comprehensively controlled or measured. Future research should incorporate longitudinal sampling and control for environmental variables to provide a clearer understanding of the microbiota’s role in respiratory health and disease.

## 5 Conclusions

The respiratory microbiota in RPBB patients is significantly more complex and dysregulated than in CG children. The reduction in beneficial genera, such as *Streptococcus* and *Granulicatella*, alongside the excessive proliferation of *Acinetobacter*, *Mycoplasma*, and other Gram-negative pathogens, may play a key role in RPBB pathogenesis. Further research on the interactions among these genera, their immunomodulatory functions, and their contributions to RPBB is essential for improving early diagnosis and prevention strategies. These studies could also guide the development of personalized therapeutic approaches.

Future research should focus on the functional characteristics and metabolic products of the RPBB-associated microbiota. The role of microbiota in host immunity and inflammatory responses remains incompletely understood. Functional genomics and metabolomics studies could provide insights into how microbial metabolic products influence immune modulation and inflammation in RPBB. Expanding sample sizes and conducting multicenter studies will enhance the generalizability and reliability of findings. These efforts may pave the way for novel strategies to improve RPBB clinical outcomes through microbiota modulation, ultimately contributing to more effective prevention and early treatment options.

## Data Availability

The original contributions presented in the study are included in the article/supplementary material, further inquiries can be directed to the corresponding author/s.
